# The presence of cystic fibrosis-related diabetes modifies the sputum microbiome in cystic fibrosis disease

**DOI:** 10.1152/ajplung.00219.2023

**Published:** 2023-12-12

**Authors:** Stanislavs Vasiljevs, Adam A. Witney, Deborah L. Baines

**Affiliations:** Institute for Infection and Immunity, St George’s, University of London, London, United Kingdom

**Keywords:** cystic fibrosis, diabetes, microbiome, sputum

## Abstract

Cystic fibrosis-related diabetes (CFRD) affects 40%–50% of adults with CF and is associated with a decline in respiratory health. The microbial flora of the lung is known to change with the development of CF disease, but how CFRD affects the microbiome has not been described. We analyzed the microbiome in sputa from 14 people with CF, 14 with CFRD, and two who were classed as pre-CFRD by extracting DNA and amplifying the variable V3-V4 region of the microbial *16S* ribosomal RNA gene by PCR. Sequences were analyzed and sources were identified to genus level. We found that the α-diversity of the microbiome using Shannon’s diversity index was increased in CFRD compared with CF. Bray Curtis dissimilarity analysis showed that there was separation of the microbiomes in CF and CFRD sputa. The most abundant phyla identified in the sputum samples were *Firmicutes* and *Proteobacteria*, *Actinobacteriota* and *Bacteroidota*, and the ratio of *Firmicutes/Bacteroidota* was reduced in CFRD compared with CF. *Pseudomonas*, *Azhorizophilus*, *Porphyromonas*, and *Actinobacillus* were more abundant in CFRD compared with CF, whereas *Staphylococcus* was less abundant. The relative abundance of these genera did not correlate with age; some correlated with a decline in FEV_1_/FVC but all correlated with hemoglobin A1C (HbA1c) indicating that development of CFRD mediates further changes to the respiratory microbiome in CF.

**NEW & NOTEWORTHY** Cystic fibrosis-related diabetes (CFRD) is associated with a decline in respiratory health. We show for the first time that there was a change in the sputum microbiome of people with CFRD compared with CF that correlated with markers of raised blood glucose.

## Graphical abstract

**Figure:**
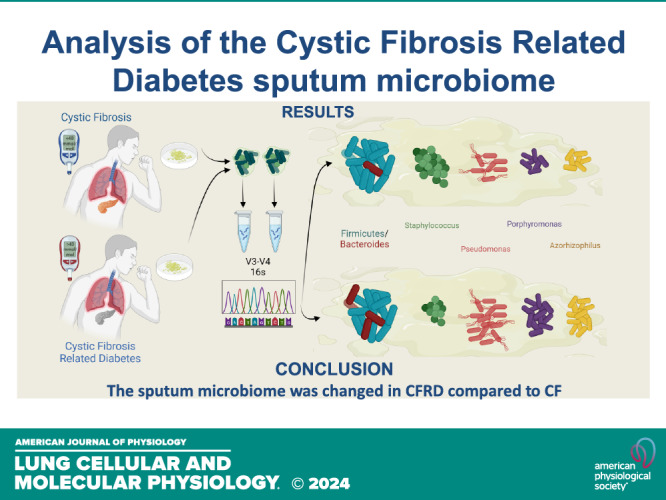

## INTRODUCTION

Cystic fibrosis (CF) is a disease of genetic origin affecting ∼100,000 people worldwide, of which 10,600 live in the United Kingdom, according to Cystic Fibrosis Trust (1, 2). Variations in the gene encoding the cystic fibrosis transmembrane regulator (CFTR), an anion channel, lead to disrupted epithelial function and the maintenance of healthy secretions in organs including the lungs and pancreas. In the lungs, defective CFTR leads to impaired mucociliary clearance, promoting inflammation, and chronic respiratory infections that eventually lead to lung failure (3). In the pancreas, CF-related pancreatic damage results in an inability to secrete sufficient insulin to maintain normal blood glucose concentration, leading to a unique type of diabetes known as cystic fibrosis-related diabetes (CFRD) which affects up to 40%–50% of adults with CF (4–6).

The microbiome of the CF lung has been investigated. Spontaneously expectorated sputum (7), bronchoalveolar lavage (BAL), protected specimen brushing (PSB) (8–11), and tissue biopsies (8, 12, 13) have all been studied. Changes in the lung microbiome profile have been associated with a decrease in lung function in individuals with CF (14, 15).

To our knowledge, no studies have specifically investigated any changes to the microbiome between individuals with CF versus individuals with CFRD (16). We hypothesized that the presence of CFRD would further change the lung microbiome of people with CF. We, therefore, carried out a small-scale study on expectorated sputum from those with CF and CFRD to examine the potential changes in the microbiome for further investigation (7, 9, 17–19).

## MATERIALS AND METHODS

### Study Design

There is evidence that the lung microbiome in people with CF (pwCF) is affected by geographical location (15). Therefore, a total of 30 spontaneously expectorated sputum samples were purchased from Manchester Allergy, Respiratory and Thoracic Surgery (ManARTS) Biobank. Fourteen sputum samples were acquired from pwCF, and 14 were acquired from people with CFRD (pwCFRD). Two samples were acquired from individuals with CF who were classified as prediabetic. Study samples were age-matched between groups as the lung microbiome and prevalence of lung pathogens change with age (1). All pwCF, due to their condition, were on antibiotic therapy. Antibiotics are known to affect the composition of the microbiome (20, 21) so we selected samples that were exposed to similar antibiotics across the groups where possible.

Individuals with CFRD were defined as having HbA1c >48 mmol/mol (>7.8 mmol/L) as per World Health Organization (WHO)-recommended guidelines (22). Written informed consent was obtained before sample collection by ManARTS biobank staff. Samples were collected voluntarily, anonymized and approval for their use in this study was granted under a biobank license (IRAS ID 180280, REC reference 15/NW/0409) in the North West Lung Research Centre at the University Hospital of South Manchester NHS Foundation Trust (UHSM). Age, sex, spirometry, HbA1C, blood glucose, and antibiotic data linked to samples were also provided (Table 1).

**Table 1.** Patient data associated with sputum samples

	CF (*n* = 14)	CFRD (*n* = 14)	Prediabetic (*n* = 2)
Male:female	9:5	8:6	2:0
Age, yr	37 ± 14	35 ± 8	36 ± 5
FVC, L	3.4 ± 1.0	3.1 ± 0.9	3.9 ± 0.2
FEV_1_, L/s	2.2 ± 0.9	1.5 ± 0.5*	2.4 ± 0.7
FEV_1_/FVC, %predicted	62.0 ± 12.0	48.6 ± 7.7**	60.4 ± 15.7
HbA1c, mmol/mol	35.4 ± 3.8	57.7 ± 6.2****	44.5 ± 2.1**
Glucose, mM	5.5 ± 1.2	10.5 ± 6.4*	5.5 ± 0.6
Medication, no. of antibiotics	2.1 ± 1.0	2.3 ± 1.1	1.5 ± 0.7

CF, cystic fibrosis; CFRD, cystic fibrosis-related diabetes. Significantly different from CF: *P < 0.05, **P < 0.01, ****P < 0.0001.

Spontaneously expectorated sputum was collected into clinic sputum sample pots with a target volume of at least 1 g. After labeling, samples were immediately transported to the laboratory and frozen within 120 min.

### DNA Extraction

Sputum samples were thawed on ice and washed with sterile phosphate-buffered saline to remove saliva. Sputum was liquified in Remel Sputasol (Thermo Fisher). Bacterial DNA from liquified sputum samples was extracted using a QIAamp DNA Microbiome Kit (Qiagen) according to the manufacturer’s guidelines. QIAamp kits utilize a pre-lysis step that digests extracellular DNA, preventing potential bias in the final analysis. Bacteria were lysed using a FastPrep-24 classic bead beating grinder and a lysis system (MPbio) at a velocity of 6.5 m/s for 45 s with a 5 min interval to ensure lysis of gram-positive bacteria. The total DNA was eluted in 50 μL of elution buffer and stored at −80°C until further use. Samples to control for potential contamination of extraction solutions were similarly processed but did not include sputum.

### Amplicon Library Preparation and Sequencing

Primers targeting the variable V3-V4 region of the microbial 16S gene were used (341 F 5′-CCTACGGGNGGCWGCAG-3′ and 805 R 5′-GACTACHVGGGTATCTAATCC-3′). The 5′ ends of the primers were tagged with barcodes unique for each sample. The PCR reaction was prepared using Q5 High-Fidelity DNA Polymerase (New England Biolabs). Each sample reaction was assembled to a total volume of 25 µL containing 25 ng of template DNA, 0.5 µL of 10 mM dNTPs, 1.25 µL of 10 µM Forward and Reverse Primers, 0.25 µL of Q5 High-Fidelity DNA Polymerase, in Q5 reaction buffer with Q5 High GC enhancer. Touchdown PCR was used for the product amplification. The PCR program consisted of 10 cycles of denaturation at 98°C for 10 s, annealing at 65°C for 30 s, with each consecutive step reducing annealing temperature by 1°C, and extension at 72°C for 45 s; this was followed by 25 cycles of denaturation at 98°C for 10 s, annealing at 54°C for 30 s, and extension at 72°C for 45 s; completing with a final extension at 72°C for 10 min. The PCR products were purified by AMPure XT beads (Beckman Coulter Genomics) and quantified by Qubit (Invitrogen). Sample quality control and sequencing were performed by LC Science (USA). Negative controls were extracted and amplified but not sequenced because they did not pass the quantitation threshold. Amplicon pools were prepared for sequencing, and the size and quantity of the amplicon library were assessed by Agilent 2100 Bioanalyzer (Agilent) and the Library Quantification Kit for Illumina (Kapa Biosciences), respectively. The libraries were sequenced on the NovaSeq PE250 platform. Raw FASTQ files were generated for further analysis.

### Data Analysis

Bioinformatic analysis was performed using Mothur v1.39.5 as per a MiSeq Standard Operating Procedure (23). SILVA v138 was used as a reference data set for alignment purposes. Operational taxonomic units (OTUs) were generated at a 97% similarity threshold. Genus-level taxonomy, α-diversity, β-diversity, and principal coordinate analysis (PCoA) loadings were generated. Downstream statistical analyses were performed using *R* statistical software and GraphPad Prism version 9. The value (*n*), represents a sputum sample from one individual.

### Data Sharing

The sequence data generated have been deposited in the European Nucleotide Archive database hosted by The European Bioinformatics Institute (www.ebi.ac.uk/ena) under BioProject Accession No. PRJEB64339.

### Statistical Analysis

α- and β-Diversity were calculated using Mothur integrated Analysis of Molecular Variance (AMOVA) and Homogeneity of Molecular Variance (HOMOVA|) analysis. HOMOVA was performed within Mothur software and is a nonparametric test based on Bartlett’s test for homogeneity of variance, which assesses whether genetic diversity within two populations is homogeneous (24). GraphPad Prism version 9 was used to perform linear regression analysis, to calculate Pearson correlation coefficients and ANOVA with post hoc Tukey’s tests or *t* tests as appropriate.

## RESULTS

**Figure 1.**
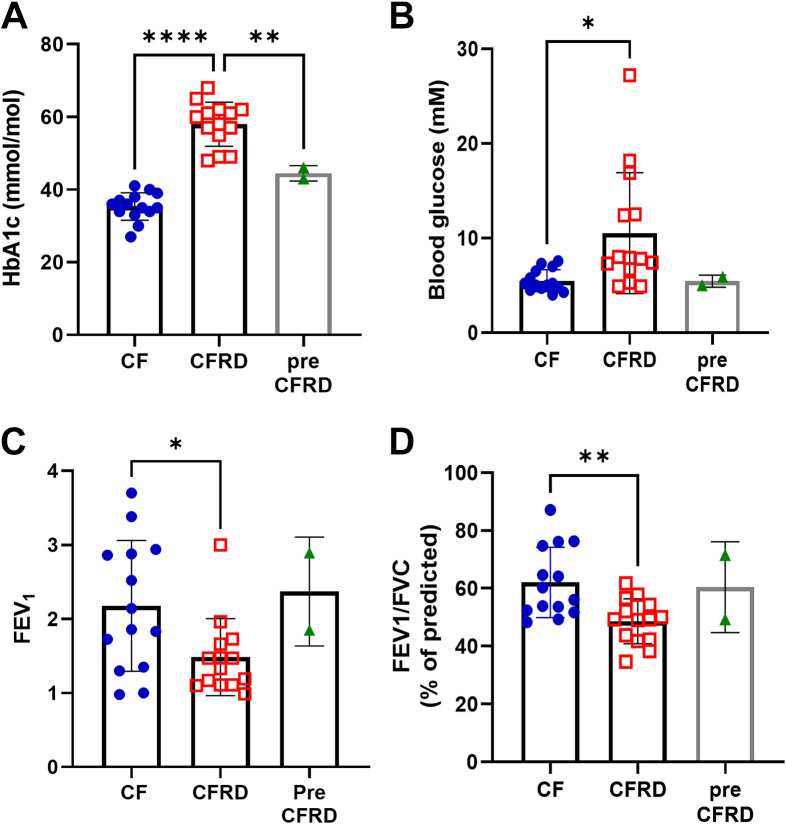
HbA1c and blood glucose were increased and lung function decreased in people with CFRD. HbA1c (*A*), blood glucose (*B*), FEV_1_ (*C*), and FEV_1_/FVC (*D*) of individuals from which sputum samples were obtained. Data points for each individual are shown: CF (CF) black circles, CFRD (CFRD) open squares, and those diagnosed as pre-CFRD (pre-CFRD) black triangles. Significant differences are shown *P < 0.05,**P < 0.01, ****P < 0.0001. CF, cystic fibrosis; CFRD, cystic fibrosis-related diabetes.

The average age of the individuals in the study was 36 yr, and patients were predominantly male in all groups. The number of antibiotics being taken by individuals was similar between those with CF and CFRD (Table 1). Most were taking azithromycin, followed by colistin or colistimethate sodium. HbA1c and fasting blood glucose concentration were significantly higher in individuals with CFRD compared with those with CF, although blood glucose concentrations were more variable, *P* < 0.0001, *n* = 14 (Table 1 and Fig. 1, *A* and *B*). Two individuals with raised HbA1c compared with the group with CF, but lower than the group with CFRD, and showed no change in fasting blood glucose were considered prediabetic (Table 1 and Fig. 1, *A* and *B*). There was no difference in FVC between the groups (Table 1) but FEV_1_ and FEV_1_/FVC were reduced in the CFRD group *P* < 0.01, *n* = 14, respectively (Table 1 and Fig. 1, *A* and *B*).

**Figure 2.**
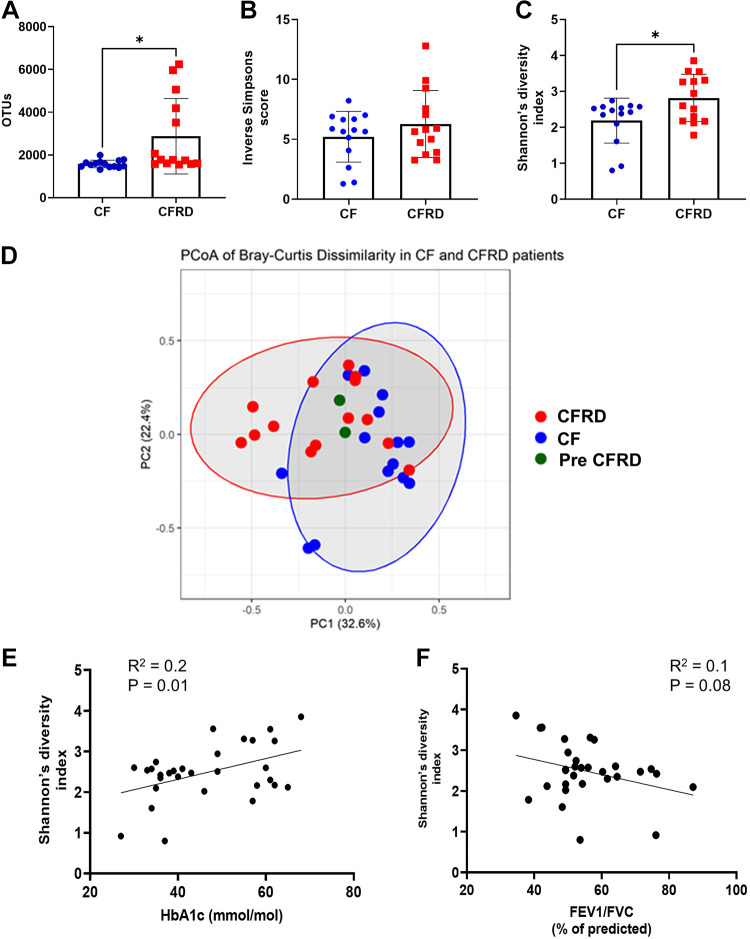
The microbiome is changed in CFRD compared with CF. OTU count (*A*), Inverse Simpsons score (*B*), Shannon’s diversity index (*C*), and PCoA plot of Bray Curtis dissimilarity of sputum microbiomes (*D*). Elipses show 95% confidence level for a multivariate t-distribution of the data. The centroids of the elipses are significantly different (*P* = 0.002, *n* = 28). Correlation of Shannon’s diversity index for HbA1c (*E*) or FEV_1_/FVC (*F*) for all individual sputa. Data points for each individual are shown: CF black/blue circles, CFRD open/red squares and those diagnosed as pre-CFRD black/green triangles. Significant differences are shown *P < 0.05. CF, cystic fibrosis; CFRD, cystic fibrosis-related diabetes; OTU, operational taxonomic unit.

There were more operational taxonomic units (OTUs) in CFRD samples compared with CF (*P* = 0.01, *n* = 14) (Fig. 2*A*). There were five samples that showed a particularly high amount of OTUs, but even without these, there was a trend toward an increase in CFRD compared with CF (*P* = 0.1, *n* = 14).

Analysis of the α-diversity of the microbiome using the Inverse Simpsons score did not indicate a difference between CF and CFRD samples (Fig. 2*B*) but the Shannon’s diversity index showed significantly increased diversity of the microbiome in CFRD samples compared with CF (Fig. 2*C*). Further analysis of β-diversity, using Bray Curtis dissimilarity, plotting the 95% confidence level for a multivariate t-distribution as ellipses and analysis of the centroids for these ellipses showed that there was separation of the microbiomes in CF versus CFRD sputa (*P* < 0.01, *n* = 28, AMOVA). The spread of the data was similar in both groups (HOMOVA). Interestingly, the two samples that were considered prediabetic, (CFRD) were positioned between these two groups suggesting a shift in the microbiome with the development of CFRD (Fig. 2*D*). There was a significant correlation between the Shannon’s diversity index and HbA1c (*P* = 0.01, *n* = 28), but not with lung function measured as % predicted FEV_1_/FVC (*n* = 28). (Fig. 2, *E* and *F*).

**Figure 3.**
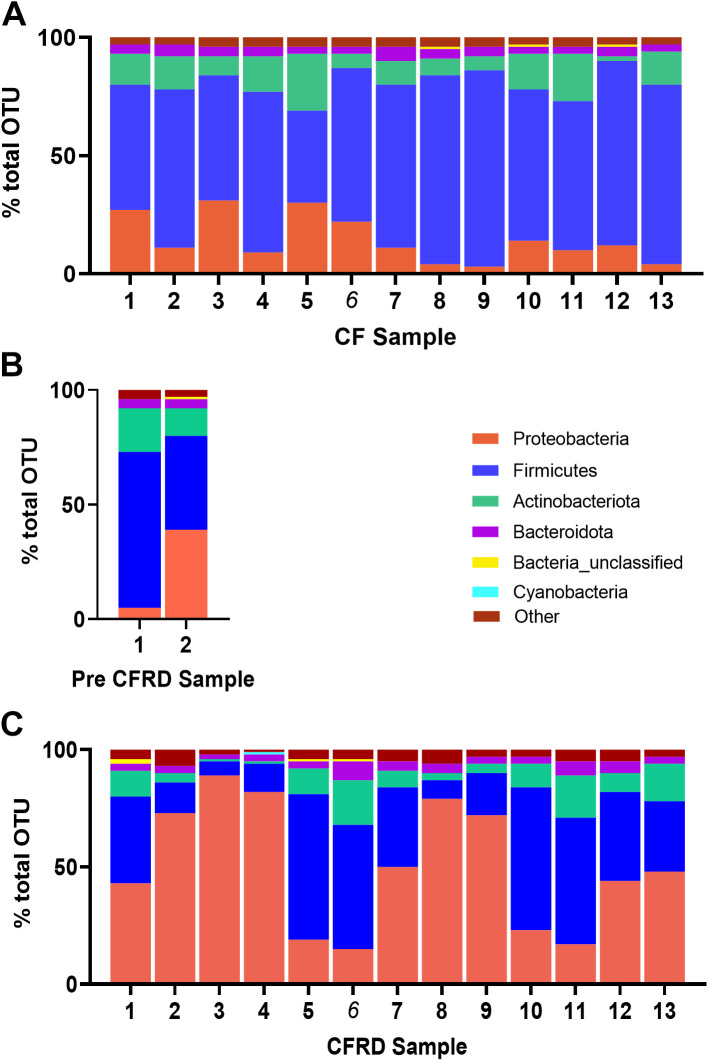
Phyla identified in the sputum microbiome of cystic fibrosis-related diabetes (CFRD) compared with cystic fibrosis (CF). The most common phyla presented as % total operational taxonomic unit (OUT) detected in sputum samples from individuals with CF (1–13; *A*), pre-CFRD (1–2; *B*), and CFRD (1–13; *C*).

**Figure 4.**
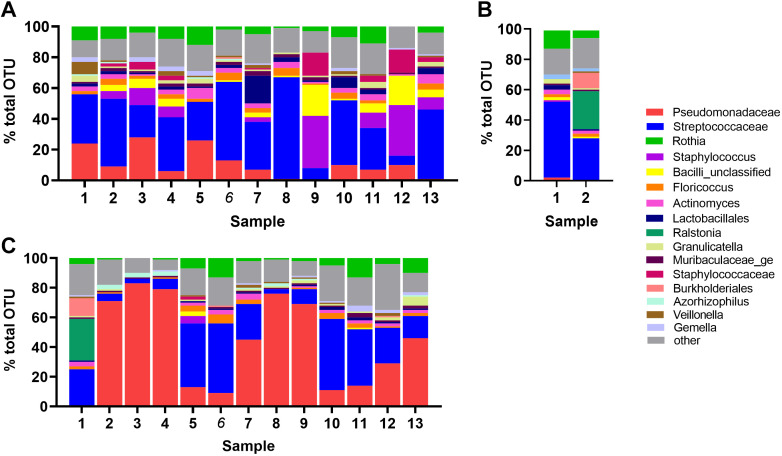
Genera identified in the sputum microbiome of CFRD compared with CF. The most common genera presented as % total OTU detected in sputum samples from individuals with CF (1–13; *A*), pre-CFRD (1–2; *B*), and CFRD (1–13; *C*). CF, cystic fibrosis; CFRD, cystic fibrosis-related diabetes; OTU, operational taxonomic unit.

The most abundant phyla identified in the sputum samples were *Firmicutes* and *Proteobacteria* (together 84%), followed by *Actinobacteriota* and *Bacteroidota* (at 9% and 4%, respectively). *Firmicutes* were consistently dominant in CF sputa. *Proteobacteria* were more dominant in the CFRD microbiome although this varied between individual sputum samples Fig. 3, *A* and *C*. One of the CF prediabetic samples was similar to CF, retaining *Firmicute* dominance, whereas the other showed codominance of *Firmicutes* and *Proteobacteria* (Fig. 3*B*). The ratio of *Firmicutes/Bacteroidota* was 16 ± 4 in CF and 8 ± 5 (*P* < 0.0001, *n* = 14) in CFRD sputa. At a genus level, *Streptococcacae* and *Staphylococcus* dominated the microbiome although *Pseudomonadacae* was present in 10/13 CF sputum samples (Fig. 4*A*). *Pseudomonadacae* were more dominant in CFRD samples, although one CFRD sample and one CF prediabetic sample exhibited increased dominance of the microbiome with *Ralstonia* and *Burkholderiales* and not *Pseudomonadacae* (Fig. 4, *B* and *C*).

**Figure 5.**
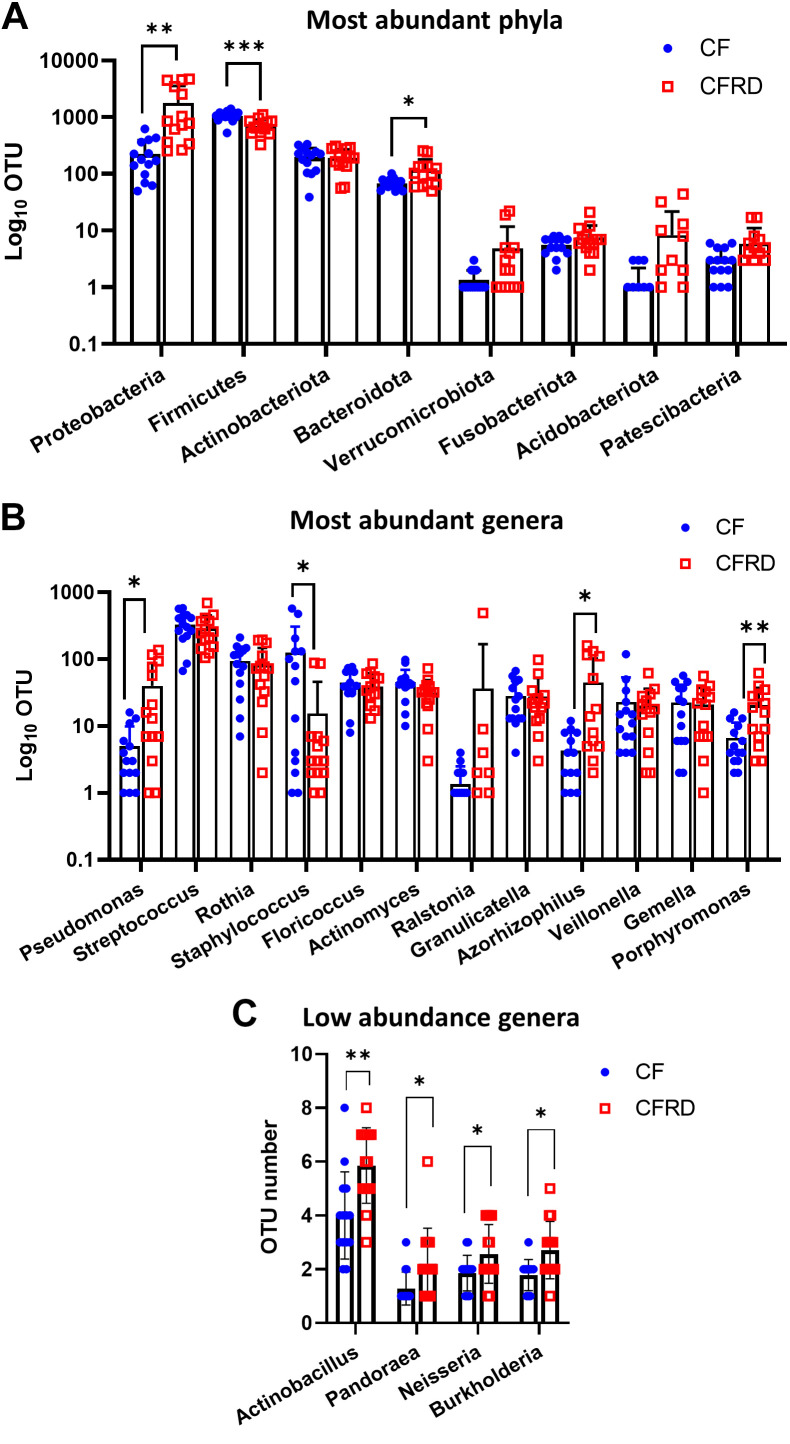
Pairwise comparison of phyla and genera in the sputum microbiome of CFRD compared with CF. Pairwise comparison of OTU for most abundant phyla (*A*), most abundant genera (*B*), and low abundance genera (*C*) identified in CF (blue-filled circles) and CFRD (red open squares) sputum samples. Significantly different from CF sputa *P < 0.05, **P < 0.01, ***P < 0.001. CF, cystic fibrosis; CFRD, cystic fibrosis-related diabetes; OTU, operational taxonomic unit.

Further analysis of the data (which included the two prediabetic samples) indicated that the number of OTUs identified as *Proteobacteria* and *Bacteroidota* significantly increased, whereas firmicutes were decreased in CFRD compared with CF sputum (*P* < 0.01, *P* < 0.05, and *P* < 0.001, respectively, *n* = 14) (Fig. 5*A*). This correlated with increased OTUs identified as *Pseudomonas*, *Azhorhizophilus*, and *Porphyromonas* and decreased *Staphylococcus* in CFRD compared with CF sputum samples (*P* < 0.05, *P* < 0.05, *P* < 0.01, and *P* < 0.05, respectively, *n* = 14) (Fig. 5*B*). Analysis of low abundance OTUs indicated that other genera such as *Actinobacillus*, *Pandorea*, *Neisseria*, and *Burkholderia/Caballeronia/Paraburkholdaria* were more common in CFRD compared with CF sputum samples (Fig. 5*C* and Supplemental Fig. S1; https://doi.org/10.6084/m9.figshare.23686677). Rhizobium, a common contaminant of microbiome extraction kits was only found at very low abundance (0.2%). Although low abundance OTUs are likely less reliable, taken together these data indicate that the number and variety of genera were increased in CFRD thus contributing to increased diversity of the microbiome in CFRD compared with CF sputum samples.

As this was a small study, we recognized that there were five CFRD sputum samples (2–4, 8, 9) with particularly high OTU of genera *Pseudomonas* that may have skewed the data. There were no differences in the parameters shown in Table 1, between these samples and others with CFRD. We therefore reanalyzed the data without these five samples. This reduced the power of the analyses and the potential of obtaining statistically significant outcomes. Nevertheless, we found that without these five samples, a trend toward decreased *Staphylococcus* in CFRD compared with CF remained (*P* = 0.08, *n* = 9). In the five samples with high *Pseudomonas* OTU alone, there was no effect on *Staphylococcus* (*P* = 0.22, *n* = 5). Although the analyses must be interpreted with care, these data indicate that high OTU numbers of genera *Pseudomonas* were not solely responsible for driving decreased OTU of genera *Staphylococcus*. We also recognized that there were four samples that showed similarity to CF samples (lower *Pseudomonas*/higher *Streptococcus*), although the clinical parameters associated with these samples remained similar to CFRD (Table 1). Removing these samples from the analysis accentuated the increase in *Pseudomonas* and lessened the decrease in *Staphylococcus* in CFRD. Thus, by leaving these samples in the analysis, the changes we show are less striking but likely a better reflection of the microbiome across a spectrum of people with CFRD. Finally, *Azorhizophilus* and *Porphyromonas* remained elevated in CFRD compared with CF in both high and low *Pseudomonas* groups tested (*P* < 0.05, *n* = 5/9) (and Supplemental Fig. S2, https://doi.org/10.6084/m9.figshare.23686686).

**Figure 6.**
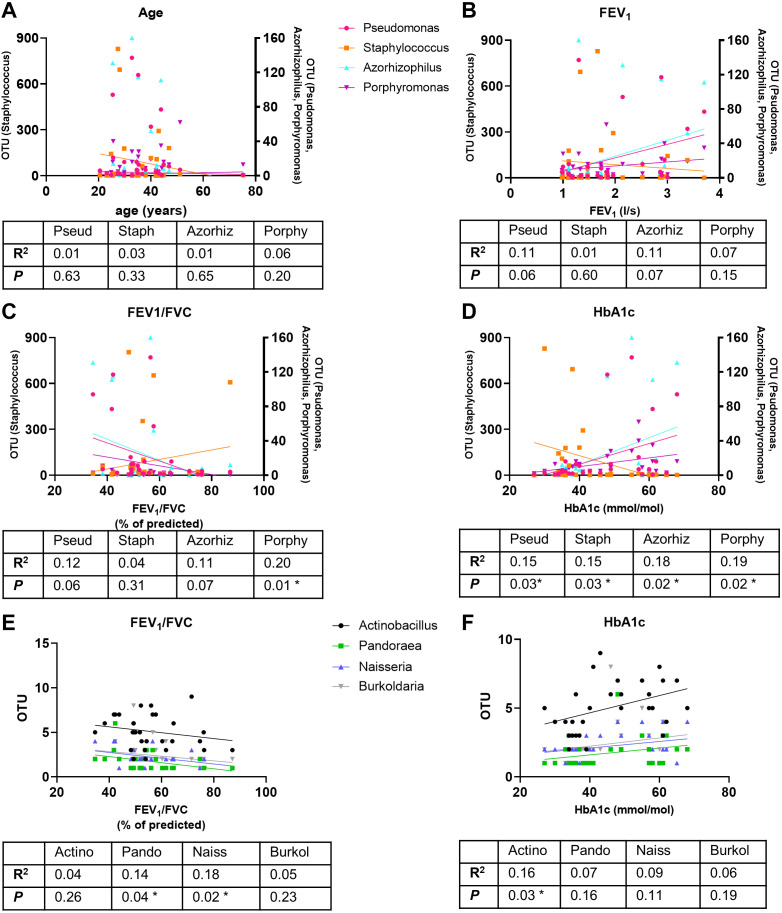
Correlation of key genera abundance with patient data. Correlation of genera *Pseudomonas, Staphylococcus*, *Azhorizophilus*, and *Porphyromonas* with age (*A*), FEV_1_ (*B*), FEV_1_/FVC (*C*), and HbA1c (*D*), and correlation of genera *Actinobacter, Pandorea, Neisseria*, and *Burkolderia* with FEV_1_/FVC (*E*) and HbA1c (*F*). The *R*^2^ and significance values are shown below each graph. Slope of the line significantly different from 0, *P < 0.05.

There was no correlation of changes in these four identified genera with age or FEV_1_. *Porphyromonas* correlated with a decline in % predicted FEV_1_/FVC (*P* < 0.01, *n* = 28) *Pseudomonas* and *Azorhizophilus* showed a similar trend but did not reach significance (*P* = 0.06 and *P* = 0.07, *n* = 28, respectively) (Fig. 6 *A*, *B*, and *C*). There was a better correlation of raised HbA1c with a decline in genera *Staphylococcus* (*P* < 0.05, *n* = 14) and an increase in *Pseudomonas, Azorhizophilus*, and *Porphyromonas* (all *P* < 0.05, *n* = 28) (Fig. 6*D*). Of the low-frequency genera identified, OTU *Pandoraea* and *Neisseria* correlated with decreased FEV1/FVC (*P* < 0.05, *n* = 28), whereas OTU *Actinobacillus* correlated with the rise in HbA1c (*P* < 0.05, *n* = 28) (Fig. 6, *E* and *F*). Thus, raised blood glucose levels in CFRD, appear to be more closely correlated to the changes in abundance of some genera that constitute the lung microbiome than age or lung function (see also Supplemental Fig. S3; https://doi.org/10.6084/m9.figshare.23686713).

## DISCUSSION

In this small study, we used sputum samples to investigate the lung microbiome in pwCF and those with CFRD. Sputum samples have been used in several other studies of the CF lung microbiome as a relatively good proxy for the identification of abundant genera throughout the lung (15, 25–30).

We identified an increase in α-diversity of the lung microbiome with CFRD compared with CF, which correlated with HbA1c but not lung function. The calculation of β-diversity (Bray-Curtis dissimilarity matrix) showed there was some commonality of the microbiomes in CF disease but also supported potential separation between the microbiome of CF and CFRD with pre-CFRD lying in the center of the two groups. Several studies have linked a decrease in α-diversity of the CF microbiome to a decrease in lung function (15, 31–34). Shannon’s index is considered a measure of richness and emphasizes rarer OTUs in its analysis compared with Simpson’s score, a measure of evenness that favors more common OTUs. Thus, we interpreted this as CFRD driving changes to rarer components of the microbiome that may have less impact on lung function than more relatively abundant components.

To understand these changes in more detail, we analyzed the phyla and genera of the microbiome. We observed that the most abundant phyla were *Proteobacteria, Firmicutes*, *Actinobacteriota*, and *Bacteroidota,* similar to that reported by others in adults with CF (35, 36). In the gut microbiome, individuals with type 1 diabetes mellitus exhibited a decrease in the *Firmicutes*/*Bacteroidota* ratio compared with nondiabetic individuals (37–39). This is consistent with our finding that the *Firmicutes*/*Bacteroidota* ratio in CFRD was almost two times lower than in CF, which indicates a similar response of the microbiome to hyperglycemia in the lung and gut.

The most abundant genera identified In the samples were consistent with the core genera observed by others in CF lungs and CF secretions (40), although we also identified *Floricoccus* and *Azorhizophilus*. *Floricoccus* are lactobacilli of the family *Streptococcaceae*, which were identified in fermented foodstuffs. Their presence in CF respiratory secretions has not been recorded although *Lactobacillus* has been identified (41). *Azorhizophilus* is a genus from the family of *Pseudomonadaceae* which has been isolated from the lungs of non-cystic fibrosis bronchiectasis (42, 43).

Of the genera identified, *Pseudomonas*, *Azhorizophilus*, *and Porphyromonas* [a strict anaerobe commonly detected in CF lungs, (9, 15)] along with many of the less common genera (e.g., *Actinobacillus)* were more abundant in CFRD compared with CF, whereas *Staphylococcus* was less abundant. These changes potentially underpin the increased richness of the microbiome we detected in CFRD. The relative abundance of these genera did not correlate with age, some correlated with a decline in FEV_1_/FVC but all correlated with HbA1c. Thus, worsening CFRD/poorly controlled blood glucose could be driving changes to the respiratory microbiome in CF.

A decrease in the relative abundance of *Staphylococcus* with an increase in *Pseudomonas* with time has been evidenced in pwCF (44). The onset of CFRD in individuals with CF increases with age, reaching 40%–50% by adulthood (4). Although we could not identify to species level, early glucose abnormalities have been shown to be associated with higher rates of *P. aeruginosa* infection (16) and it is well-documented that CFRD is associated with higher odds of being chronically infected with *P. aeruginosa* (45). As we did not find any relationship with age, we suggest that the development of CFRD and increased HbA1c plays a key part in this transition. Early colonizing isolates of *P. aeruginosa* show strong antagonism toward *S. aureus* in CF lungs, although this is not sustained in chronic infections and both can exist together (46–50). It is possible that development of hyperglycemia in CFRD alters the relationship between the two genera. For example, in artificial sputum medium, we showed that the presence of glucose can alter the growth dynamics of *P. aeruginosa and S. aureus* in coculture (51).

Diabetes is a complex disease, with endocrine, inflammatory, and nutritional components. Nevertheless, in vitro and in vivo, elevated glucose was shown to be associated with increased growth of *P. aeruginosa* and *S. aureus* (45, 52). Drugs that lowered blood glucose in diabetic mice or restricted glucose movement across the airway epithelium reduced the luminal growth of *P. aeruginosa* suggesting that glucose itself has a role in promoting changes associated with CFRD (53–55).

*Pseudomonas* infection is associated with respiratory decline in pwCF. The roles of *Actinobacillus* (a traditional CF pathogen associated with respiratory secretions) and *Azorhizophilus* in respiratory health are unclear (40, 56–58). There is also little evidence whether increased abundance of *Actinobacillus* in CFRD lungs is associated with lung function decline or contributes to disease progression. However, elevation of some species of *Porphyromonas* has been associated with a more rapid decline in lung function in pwCF consistent with observations in studies by Webb in 2022 (59) and Acosta in 2018 (60).

In conclusion, this small study demonstrated that the sputum microbiome was changed in CFRD compared with CF. The ratio of *Firmicutes* to *Bacteriodes* was significantly reduced in CFRD compared with CF. Changes in the relative abundance of key genera correlated with HbA1c. Although we cannot exclude that other contributory health factors in individuals with higher HbA1c could influence the microbiome, we suggest that further study of the CFRD microbiome, particularly in response to treatment with insulin/CFTR modulators, would aid the development of precision medicine to reduce respiratory decline in pwCFRD.

## DATA AVAILABILITY

The sequence data generated have been deposited in the European Nucleotide Archive database hosted by The European Bioinformatics Institute (https://www.ebi.ac.uk/ena/browser/view/PRJEB64339).

## SUPPLEMENTAL DATA

10.6084/m9.figshare.23686677 Supplemental Fig. S1: https://doi.org/10.6084/m9.figshare.23686677.

10.6084/m9.figshare.23686686 Supplemental Fig. S2: https://doi.org/10.6084/m9.figshare.23686686.

10.6084/m9.figshare.23686713 Supplemental Fig. S3: https://doi.org/10.6084/m9.figshare.23686713.

## GRANTS

This work was funded by a Medical Research Council - Doctoral Training Partnership (MRC-DTP) studentship to S. Vasiljevs.

## DISCLOSURES

No conflicts of interest, financial or otherwise, are declared by the authors.

## AUTHOR CONTRIBUTIONS

D.L.B. conceived and designed research; S.V. performed experiments; S.V. and A.A.W. analyzed data; S.V. and A.A.W. interpreted results of experiments; S.V. prepared figures; S.V. and D.L.B. drafted manuscript; A.A.W. and D.L.B. edited and revised manuscript; S.V., A.A.W., and D.L.B. approved final version of manuscript.
